# Transcriptome Analysis of Two Species of Jute in Response to Polyethylene Glycol (PEG)- induced Drought Stress

**DOI:** 10.1038/s41598-017-16812-5

**Published:** 2017-11-29

**Authors:** Zemao Yang, Zhigang Dai, Ruike Lu, Bibo Wu, Qing Tang, Ying Xu, Chaohua Cheng, Jianguang Su

**Affiliations:** 1 Germplasm lab, Institute of Bast Fiber Crops, Chinese Academy of Agricultural Sciences, Changsha, Hunan China; 2 Hunan Biological And Electromechanical Polytechnic, Changsha, Hunan China

## Abstract

Drought stress results in significant crop yield losses. Comparative transcriptome analysis between tolerant and sensitive species can provide insights into drought tolerance mechanisms in jute. We present a comprehensive study on drought tolerance in two jute species—a drought tolerant species (*Corchorus olitorius* L., GF) and a drought sensitive species (*Corchorus capsularis* L., YY). In total, 45,831 non-redundant unigenes with average sequence length of 1421 bp were identified. Higher numbers of differentially expressed genes (DEGs) were discovered in YY (794) than in GF (39), implying that YY was relatively more vulnerable or hyper-responsive to drought stress at the molecular level; the two main pathways, phenylpropanoid biosynthesis and peroxisome pathway, significantly involved in scavenging of reactive oxygen species (ROS) and 14 unigenes in the two pathways presented a significant differential expression in response to increase of superoxide. Our classification analysis showed that 1769 transcription factors can be grouped into 81 families and 948 protein kinases (PKs) into 122 families. In YY, we identified 34 TF DEGs from and 23 PK DEGs, including 19 receptor-like kinases (RLKs). Most of these RLKs were downregulated during drought stress, implying their role as negative regulators of the drought tolerance mechanism in jute.

## Introduction

Crop production is greatly affected by drought, one of the major abiotic stresses that cause significant yield loss^1,2^. Drought is further aggravated by population growth, global scarcity of water resources, and climate abnormalities. Therefore, it is of crucial importance to clarify the mechanisms of drought stress tolerance in crops in order to solve or alleviate the problems caused by this stress^2^.

Plant response to drought stress is a complex biological process involving physiological, biochemical, and molecular changes. During this process, the expression profiles of a large number of genes are altered; these genes relate mainly to two classes of genes. The first class includes genes encoding ‘effector proteins’, which directly protect plants from abiotic stress including membrane protein genes; genes involved in biosynthesis of various osmoprotectants; photosynthesis-related genes; genes involved in growth and development; chaperones-encoding genes; and genes encoding detoxification enzymes^3,4^ etc. The second class of genes encode ‘regulatory proteins’ that regulate the expression of downstream target genes in the stress response^3^ and include protein kinases (PKs), such as mitogen-activated PKs, ribosomal PKs, and receptor PKs^1^, and transcription factors (TFs), such as AP2/ERF, AREB/ABF, bZIP, DREB, MYC/MYB, and WRKY^3,5,6^.

As well, many plant metabolism pathways participate in response to abiotic stress, such as photosynthetic, phenylalanine metabolism, peroxidase, MAPK signalling pathways, etc. A delicate balance between the multiple pathways is necessary for plant growth and development^7^. However, the balance might be be broken when the plant is underlying abiotic stress. For example, ROS, such as O^−^_2_, H_2_O_2_, etc. can be used as signaling molecule when keeping at low levels under normal conditions. Production and balance of ROS is related to photosynthetic, phenylalanine metabolism, peroxidase pathways^8,9^.Underlying stress, the electrons of photosynthetic pathway leak to O_2_ and result in generation of a mass of ROS. In order to cope with the crisis, numerous effector genes involved in phenylalanine metabolism and peroxidase pathways present differential expression, such as ferulic acid^10^, sinapic acid^11^, isocitrate dehydrogenase (ICDH), superoxide dismutase (SOD), catalase (CAT)^7^, etc. If these pathways are able to reconstruct the new balance and preserve ROS at a relativly low level, the tissues and cells may be prevented from damage and death because of oxidation.

Advancements in high-throughput sequencing technology resulted in cost reductions, rendering transcriptome sequencing the most direct and effective way to explore stress resistance mechanism in plants. Recent studies^12,13^ have shown that comparative analysis of transcriptomes between a tolerant and a sensitive genotype can effectively elucidate the molecular mechanism of abiotic stress. After cotton, jute (*Corchorus* spp.) is the second most important global biodegradable natural fibre crop. It is a diploid annual crop (2*n* = 14) distributed in the tropics, subtropics, and warm temperate regions of the world (mainly in Asia and Africa)^14^. It includes two cultivated species, *C. capsularis* L., a drought sensitive species, and *C. olitorius* L., a drought tolerant species. Recently, the demand for jute has increased worldwide because of its broad-spectrum application and eco-friendly characteristics^15,16^.

Several studies on drought tolerance in jute plants focused mainly on drought-resistance evaluation of germplasm^17,18^ and morphological^19^, physiological^20^, and biochemical^21^ changes during the response period. However, to our knowledge, to date only Sawarkar *et al*.^22^ and Das *et al*.^23^ have studied the genetics of jute under drought conditions, and a comprehensive and high-throughput study on drought tolerance is yet to be conducted. Here, we carried out transcriptome sequencing in *C. capsularis* and *C. olitorius* exposed to drought stress to explore the drought resistance mechanism in these cultivated species.

## Results

### Transcriptome sequencing and assembly

Twelve GF and YY samples were used for transcriptome sequencing and analysis, generating a total of 608,395,184 raw reads (Table 1). After quality control of these reads, 587,249, 098 (96.52%) clean reads were obtained from the raw data with an average GC content of 43.37% and Q20 average bases quality score of 97.14%, accounting for 88.1 Gb of sequencing data, which were used for *de novo* assembly of transcriptome. A total of 123,327 transcripts and 45,831 non-redundant unigenes were assembled using all clean reads. The length of the transcripts and unigenes varied from 201 to 15,890 bp, with averages of 1716 and 1421 bp, respectively (Table 2). The sequence raw reads and obtained in the present study are available in the NCBI Sequence Read Archive (SRA) under BioProject number PRJNA378897.

**Table 1:** Details of the raw data and clean data of twelve transcriptomes from drought-tolerant *Corchorus olitorius* (GF) and drought-sensitive *C. capsularis* (YY).

Species	Raw reads		Clean reads		Clean bases (Gb)		Mapped reads avg (%)		Q20 avg (%)	GC avg (%)
	Control	Drought	Control	Drought	Control	Drought	Control	Drought		
GF	147,199,898	161,498,660	142,004,554	156,115,186	21.3	23.42	74.08	74.12	97.14	43.37
YY	157,609,856	142,086,770	152,431,084	136,698,274	22.88	20.5	76.87	74.29		
total	608,395,184		587,249,098 (96.52%)		88.1		—	—

**Table 2:** Assembly output statistics of all clean data using Trinity assembler.

	Min Length	Mean Length	Max Length	N50	Number	Total Nucleotides
Transcripts	201	1716	15,573	2327	123,327	211,649,647
Unigenes	201	1421	15,573	2131	45,831	65,148,415

### Functional annotation of unigenes

Sequence alignments of all 45,831 unigenes to the NCBI non-redundant (Nr), NCBI nucleotide (Nt), Kyoto Encyclopedia of Genes and Genomes (KEGG) Orthology (KO), gene ontology (GO), eukaryotic ortholog group (KOG), protein family (Pfam), and SwissProt databases revealed that 30,799 (75.79%) unigenes were successfully annotated in at least one databases and 6,520 (14.22%) unigenes were annotated in all seven databases. The largest number of unigenes (30, 799; 67.2%) was aligned to the Nr database, whereas more than 50% of unigenes were aligned to each the SwissProt, Pfam, GO, and Nt databases. The lowest number of unigenes (12,305; 26.84%) was annotated in the KO database (Supplemental Fig. 1).

Meanwhile, unigenes within GO annotation were classified into 46 terms involved in cellular components, biological processes, and molecular function (Supplemental Fig. 2). Comparison to the KEGG database revealed that the successfully annotated unigenes were assigned to 280 KEGG pathways, which were grouped into 32 classifications based on pathway hierarchy 2 (Supplemental Fig. 3). Unigenes successfully annotated in KOG were aligned to 26 KOG classifications (Supplemental Fig. 4).

### Differential gene expression in response to polyethylene glycol treatment

**Figure 1:**
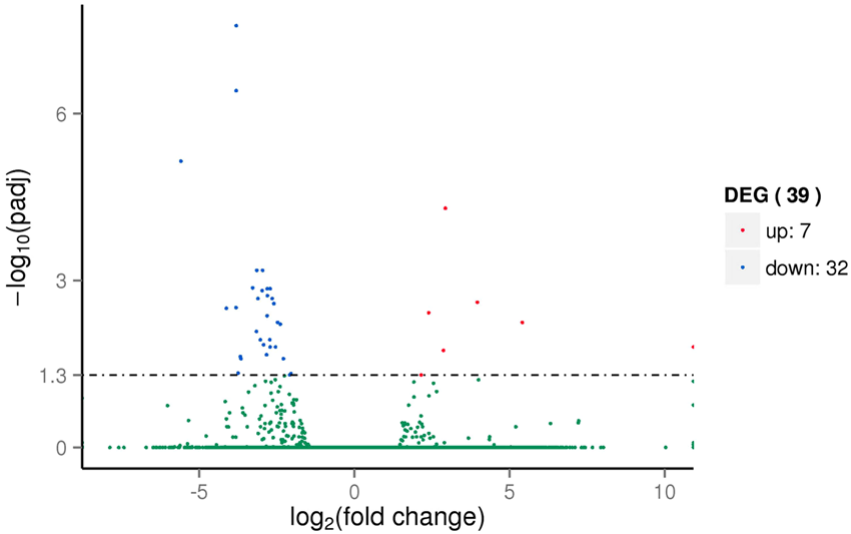
Analysis of differentially expressed unigenes in GFD (GF drought-stressed) vs. GFC (GF control).

**Figure 2:**
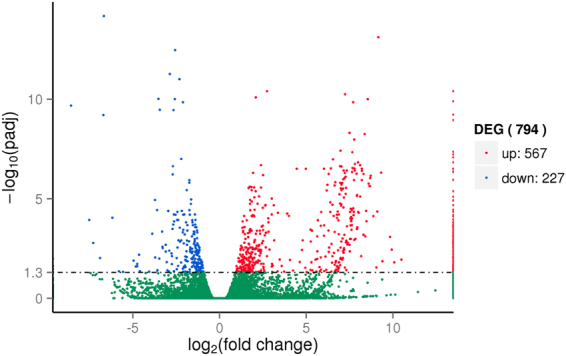
Analysis of differentially expressed unigenes in YYD (YY drought-stressed) vs. YYC (YY control).

The analysis of GF drought-stressed (GFD) vs. GF control (GFC) and YY drought-stressed (YYD) vs. YY control (YYC) revealed 39 (Fig. 1) and 794 (Fig. 2) significantly differentially expressed unigenes (DEGs) in GF and YY, respectively (Supplementary file 1). Of those, 7 and 567 DEGs were upregulated and 32 and 227 were down-regulated in GFD vs. GFC and YYD vs. YYC, respectively. Between GF and YY, there were only 7 common DEGs, all of which were down-regulated.

### GO and KEGG analysis of differentially expressed unigenes

**Figure 3:**
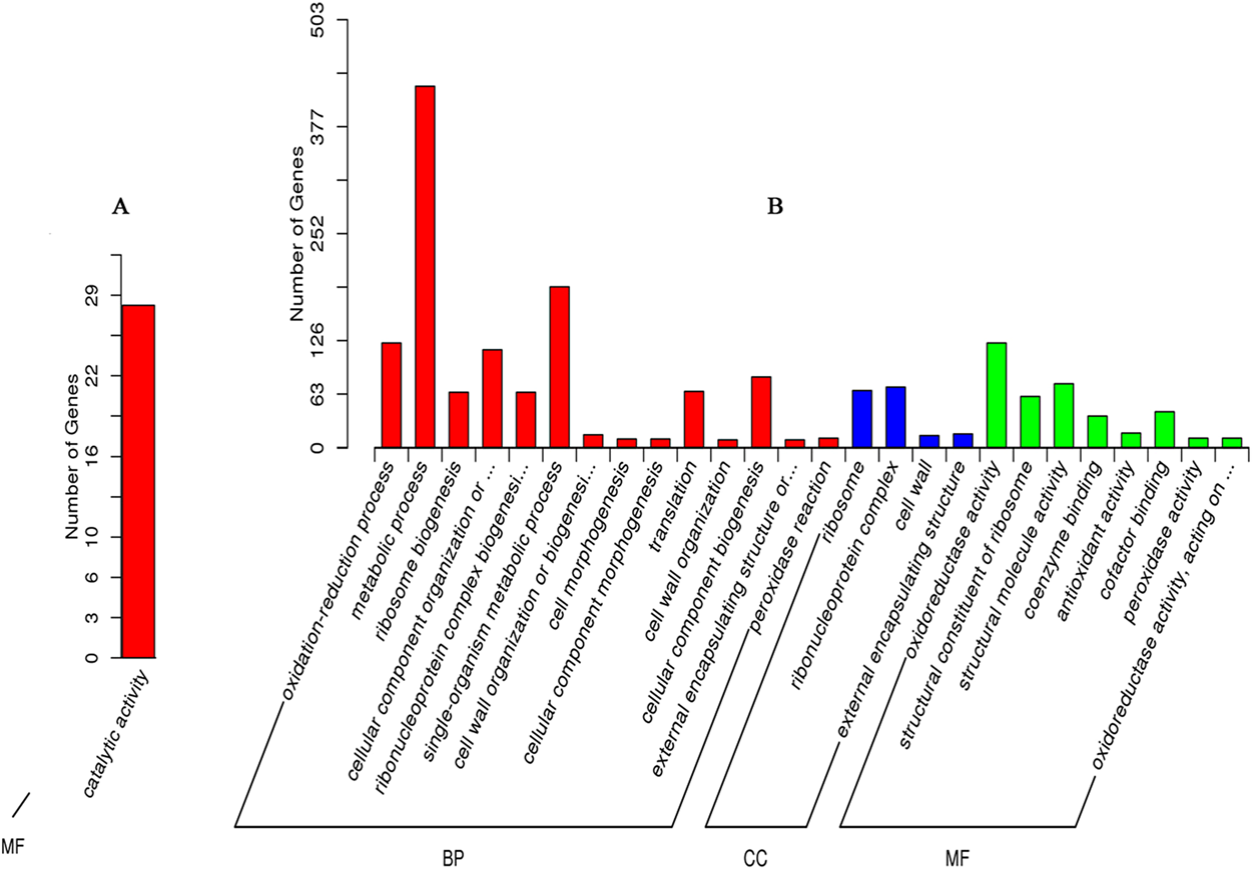
Gene ontology analysis showed that the differentially expressed unigenes (DEGs) enrichment categories in GFD (GF drought-stressed) vs. GFC (GF control) (**A**) and in YYD(YY drought-stressed) vs. YYC (YY control) for biological process (BP) (**B**), cellular component (CC) (**B**), and molecular function (MF) (**B**).

We carried out GO annotation of 39 and 794 DEGs identified in GF and YY, respectively. In the drought tolerant species, only 28 genes were significantly enriched in catalytic activity under the molecular function (Fig. 3A). In the drought sensitive species, under the biological process (BP) category, metabolic process, translation, oxidation-reduction process, cell wall organisation or biogenesis, cell morphogenesis, and ribosome biogenesis etc. were prominently represented (Fig. 3B). Under the cellular component (CC) category, a large number of unigenes were enriched in cell wall and ribosome etc. (Fig. 3B), whereas for the molecular function (MF) category, oxidoreductase activity, peroxidase activity, structural constituent of ribosome, and coenzyme binding etc. were significantly enriched (Fig. 3B).

The KEGG pathway analysis showed that DEGs in YY were mainly located in ribosome, carbon metabolism, protein processing in endoplasmic reticulum, oxidative phosphorylation, biosynthesis of amino acids, phenylpropanoid biosynthesis, glycolysis/gluconeogenesis, glyoxylate and dicarboxylate metabolism, carbon fixation in photosynthetic organisms, MAPK signalling pathway, lysosome, citrate cycle (TCA cycle), plant–pathogen interaction, phenylalanine metabolism, and arginine and proline metabolism (Table 3). In contrast, in GF, only the fatty acid metabolism pathway was enriched for three unigenes.

**Table 3:** Main KEGG enriched pathway of differentially expressed genes (DEGs) in drought-sensitive *Corchorus capsularis*.

Pathway	KEGG pathway ID	DEGs number
Ribosome	ko03010	74
Carbon metabolism	ko01200	24
Protein processing in endoplasmic reticulum	ko04141	24
Oxidative phosphorylation	ko00190	20
Biosynthesis of amino acids	ko01230	16
Phenylpropanoid biosynthesis	ko00940	14
Glycolysis/Gluconeogenesis	ko00010	14
Glyoxylate and dicarboxylate metabolism	ko00630	13
Carbon fixation in photosynthetic organisms	ko00710	13
MAPK signalling pathway	ko04010	13
Lysosome	ko04142	13
Citrate cycle (TCA cycle)	ko00020	12
Plant-pathogen interaction	ko04626	12
Phenylalanine metabolism	ko00360	11
Arginine and proline metabolism	ko00330	11
Pyruvate metabolism	ko00620	9
Endocytosis	ko04144	9
Valine, leucine and isoleucine degradation	ko00280	8
Starch and sucrose metabolism	ko00500	8
RNA transport	ko03013	8
Pentose and glucuronate interconversions	ko00040	7
Peroxisome	ko04146	7
Cysteine and methionine metabolism	ko00270	7

### Differential expression of TFs

A total of 1769 unigenes encoding TFs were classified into 81 TF families (Supplementary file 2). The highest number of unigenes was in the MYB family, with 143 unigenes, followed by AP2-EREBP, Orphans, bHLH, and NAC families, each including more than 80 unigenes. In YY under drought stress, 34 DEGs encoding TFs belonged to 20 TF families: AP2-EREBP, MYB, C2C2-Dof, HB, bHLH, TCP, CCAAT, Orphans, ARID, HSF, C2H2, bZIP, C2C2-CO-like, MBF1, GRAS, C3H, BBR/BPC, NAC, HMG, and TRAF (Table 4). Of these, AP2-EREBP included the highest number of DEGs (5), followed by MYB, C2C2-Dof, and HB.

**Table 4:** Expression and function annotation of 34 differentially expressed genes (DEGs) encoding transcription factors (TFs) in drought-sensitive *Corchorus capsularis*.

Unigenes	TF family	log_2_(FC)	Function annotation
c81341_g1	AP2-EREBP	−1.1847	regulation of transcription, DNA-dependent
c88904_g1	AP2-EREBP	1.3936	regulation of transcription, DNA-dependent//viral infectious cycle//signal transduction
c83083_g1	AP2-EREBP	1.7811	regulation of transcription, DNA-dependent
c87442_g1	AP2-EREBP	2.3182	sensory perception of chemical stimulus//regulation of transcription, DNA-dependent
c80754_g1	AP2-EREBP	2.8227	pathogenesis//regulation of transcription, DNA-dependent
c84857_g2	ARID	−1.4303	tRNA aminoacylation for protein translation
c88699_g1	BBR/BPC	−1.2835	metabolic process//photosynthesis
c83837_g1	bHLH	−1.7142	—
c82269_g1	bHLH	−1.4277	—
c81311_g1	bZIP	1.7312	DNA replication//autophagy//regulation of transcription, DNA-dependent//cell cycle
c81026_g2	C2C2-CO-like	−2.2776	—
c81214_g4	C2C2-Dof	−1.3781	pathogenesis//oxidation-reduction process//ion transport//regulation of transcription
c89585_g4	C2C2-Dof	−1.08	regulation of transcription, DNA-dependent
c79713_g4	C2C2-Dof	1.7456	regulation of transcription, DNA-dependent
c82698_g1	C2H2	3.0106	—
c89112_g2	C3H	−1.3664	tRNA processing//oxidation-reduction process//carotenoid biosynthetic process
c78769_g3	CCAAT	1.0078	DNA-dependent transcription, initiation
c77173_g1	CCAAT	7.0214	—
c85510_g1	GRAS	−1.2846	regulation of transcription, DNA-dependent
c80964_g1	HB	−1.8847	regulation of transcription, DNA-dependent
c88433_g2	HB	−1.7419	regulation of transcription, DNA-dependent
c87626_g1	HB	−1.2914	glycolipid transport//barrier septum assembly//regulation of transcription
c76701_g2	HMG	10.065	regulation of transcription, DNA-dependent//transport
c86122_g1	HSF	1.5553	regulation of transcription, DNA-dependent//spindle assembly
c85671_g1	MBF1	2.1036	oxidation-reduction process//metabolic process
c80089_g1	MYB	−2.1779	—
c89680_g1	MYB	1.3701	—
c80040_g2	MYB	1.7617	transport//drug transmembrane transport//transmembrane transport//drug transport
c76176_g1	NAC	2.3436	oxidation-reduction process//regulation of transcription, DNA-dependent
c89867_g2	Orphans	−1.4272	—
c83081_g1	Orphans	1.6443	phosphorelay signal transduction system
c80837_g1	TCP	−1.3654	pantothenate biosynthetic process//malate transport//oxidation-reduction process
c79759_g2	TCP	−1.1567	sucrose metabolic process//starch metabolic process//galactose metabolic process
c68683_g1	TRAF	5.6375	modulation by virus of host morphology or physiology

FC: fold change.

### Protein kinases in response to drought stress

Eukaryotic genomes have a large number of PK genes and play an important role in phosphorylation events that activate and inactivate the downstream signalling pathway. A total of 948 PK genes, belonging to 122 PK families, were identified (Supplementary file 3). The most abundant family was RLK-Pelle_DLSV with 93 members, followed by RLK-Pelle_LRR-XI-1 with 53 members. The families RLK-Pelle_DLSV, RLK-Pelle_LRR-XI-1, RLK-Pelle_L-LEC, RLK-Pelle_CrRLK1L-1, CAMK_CDPK, RLK-Pelle_SD-2b, RLK-Pelle_LRR-III, RLK-Pelle_LRR-XII-1, RLK-Pelle_RLCK-VIIa-2, CAMK_CAMKL-CHK1, and TKL-Pl-4 each included more than 20 members. 15 families included 23 DEGs in YY, and the highest number of DEGs (19) was assigned to the receptor-like kinases (RLK) class. Most of the DEGs were downregulated by drought stress (Table 5).

**Table 5:** Analysis of protein kinase differentially expressed genes (DEGs) in drought-sensitive *Corchorus capsularis* (YY) under drought stress (YYD) and normal (YYC) conditions.

PK families	DEGs	LogFC (YYD vs. YYC)	Annotation
AGC-Pl	c77309_g1	5.214	AGC protein kinase
AGC_RSK-2	c88811_g2	−1.268	Phototropin 1 isoform 1
RLK-Pelle_CR4L	c89074_g1	−1.7142	Crinkly 4
RLK-Pelle_CrRLK1L-1	c84515_g1	−1.0488	Malectin/receptor-like protein kinase family protein
RLK-Pelle_CrRLK1L-1	c84566_g1	−2.2044	Malectin/receptor-like protein kinase family protein
RLK-Pelle_CrRLK1L-1	c86137_g3	−1.4011	Kinase superfamily protein, putative isoform 1
RLK-Pelle_DLSV	c83696_g1	−3.7212	S-locus lectin protein kinase family protein
RLK-Pelle_DLSV	c84899_g1	−1.682	Serine/threonine kinases, protein kinases, ATP binding, sugar
RLK-Pelle_DLSV	c89207_g1	−2.2017	Leucine-rich repeat transmembrane protein kinase
RLK-Pelle_DLSV	c89313_g1	−1.5947	Cysteine-rich RLK 10
RLK-Pelle_DLSV	c90903_g5	−2.4626	S-locus lectin protein kinase family protein
RLK-Pelle_L-LEC	c84451_g1	−2.6991	Kinase, putative
RLK-Pelle_LRR-III	c89009_g1	−1.2063	Leucine-rich repeat protein kinase family protein isoform 1
RLK-Pelle_LRR-VI-1	c89569_g1	−1.4033	Leucine-rich repeat protein kinase family protein isoform 1
RLK-Pelle_LRR-VII-1	c89862_g1	−1.9284	Leucine-rich repeat protein kinase family protein isoform 1
RLK-Pelle_LRR-VIII-1	c88539_g1	1.6051	LRR receptor-like serine/threonine-protein kinase, putative
RLK-Pelle_LRR-XI-1	c88948_g1	−1.1307	HAESA-like 1 isoform 1
RLK-Pelle_LRR-XII-1	c90920_g2	−1.619	Leucine-rich repeat protein kinase family protein, putative
RLK-Pelle_LysM	c88139_g2	2.2853	PREDICTED: chitin elicitor receptor kinase 1-like
RLK-Pelle_SD-2b	c85931_g1	1.7428	Receptor protein kinase 1
RLK-Pelle_SD-2b	c86648_g4	−3.273	Receptor protein kinase 1
STE_STE11	c77570_g1	1.8479	Mitogen-activated protein kinase kinase kinase 15
STE_STE11	c85006_g6	−2.6717	PREDICTED: mitogen-activated protein kinase kinase kinase 3-like

FC: fold change.

### A comprehensive scavenging pathway of ROS

**Figure 4:**
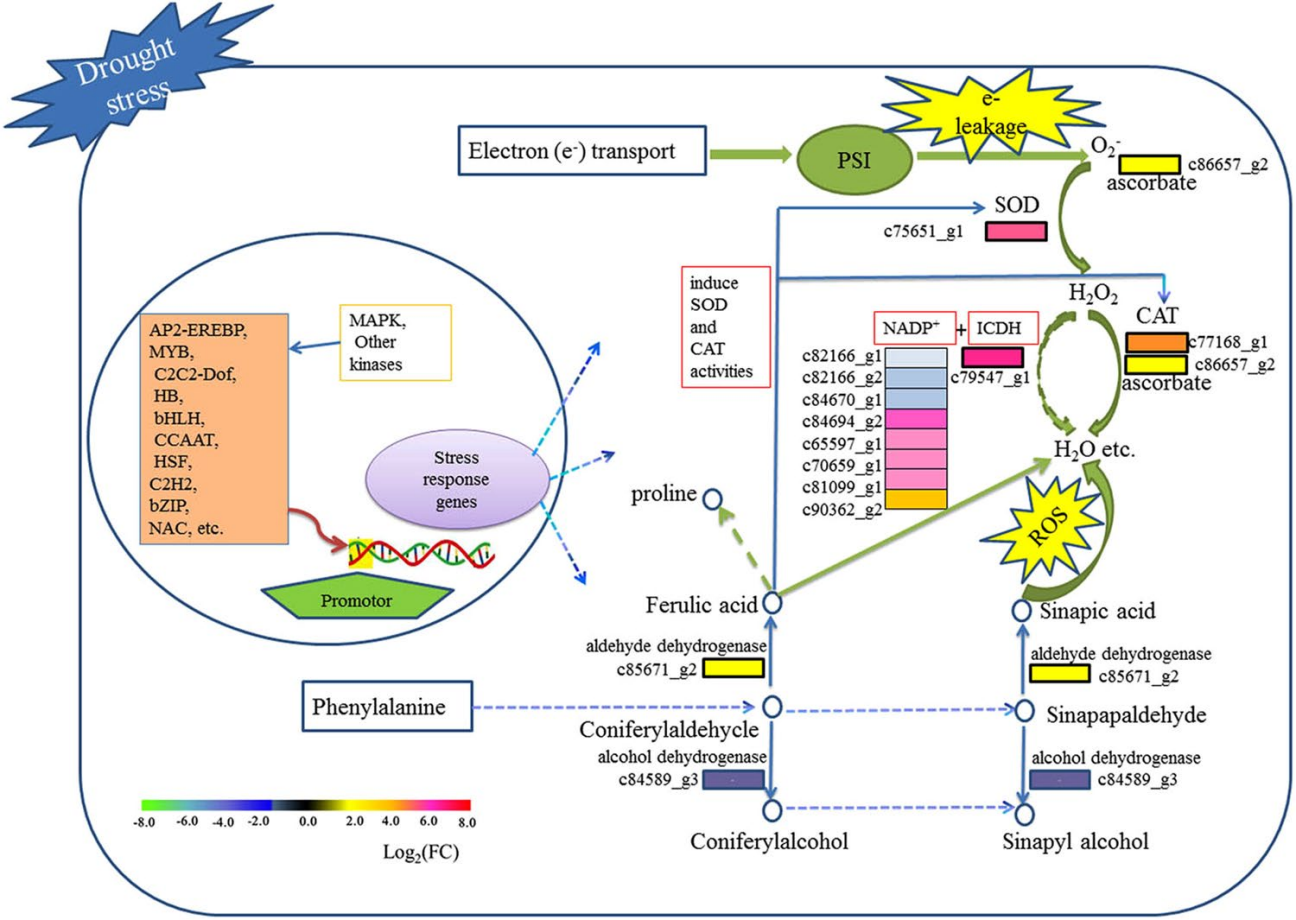
Fourteen differentially expressed unigenes (DEGs) involved in phenylpropanoid biosynthesis and peroxisome pathway showed a comprehensive pathway in response to increase of superoxide. PSI: photosystem I, SOD: superoxide dismutase, ICDH: isocitrate dehydrogenase, CAT: catalase, ROS: reactive oxygen species.

The accumulation of ROS can damage to DNA, RNA, proteins and lipids and then result in disrupting normal metabolism and even tissues and cell death. In order to cope with the toxicity of ROS, plants have evolved an array of efficient cooperative system included enzymatic and nonenzymatic antioxidants and involved in multiple metabolic pathway. In YY, under drought stress, the two main pathways, phenylpropanoid biosynthesis and peroxisome pathway, significantly involved in scavenging of ROS, and 14 unigenes in the two pathways presented a significant differential expression in response to increase of superoxide (Fig. 4): one SOD (upregulated, 104.42 fold change), one ascorbate (upregulated, 3.26 fold change), one CAT unigene (upregulated, 28.89 fold change), one ICDH (upregulated, 147.54 fold change), eight NADP+ (five upregulated, 8.37~83.87 fold change; three dowregulated, −2.35~−2.84), one aldehyde dehydrogenase (upregulated, 3.61 fold change) and one alcohol dehydrogenase (downregulated, −2.32 fold change).

### SNP marker identification

**Figure 5:**
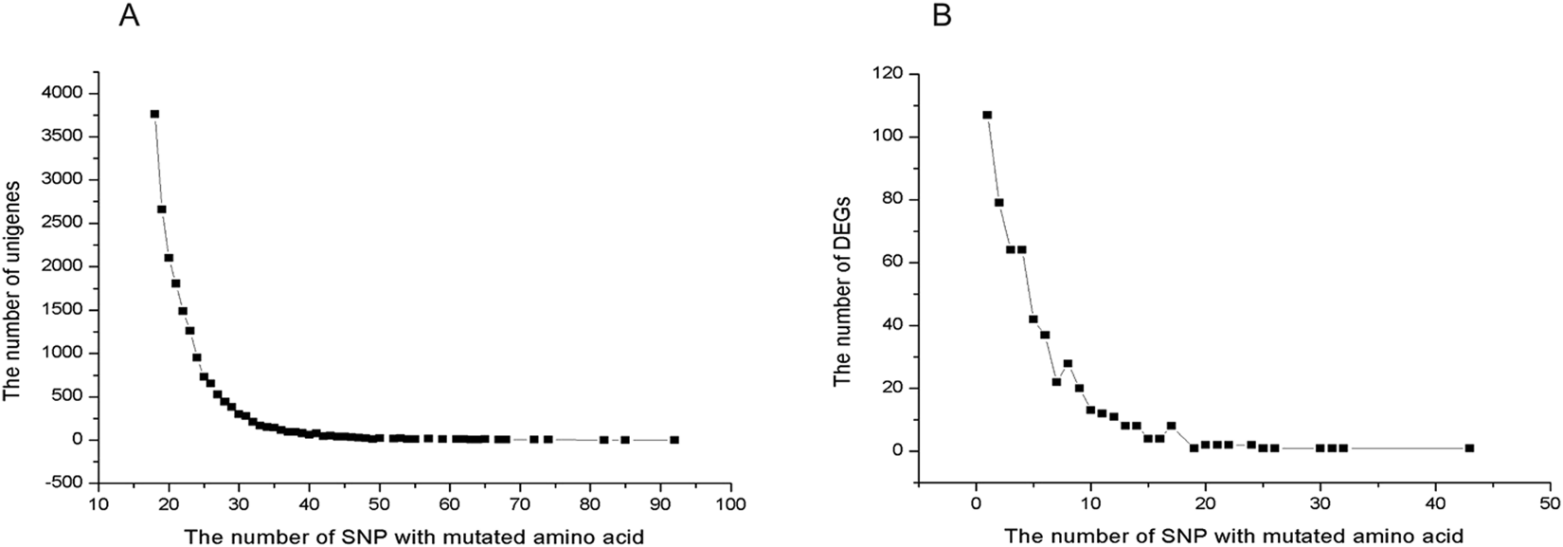
Satistics of SNP sites with mutated amino acid and unigenes included those SNP sites (**A**); Satistics of differentially expressed unigenes (DEGs) included SNP sites with mutated amino acid and those SNP sites (**B**).

In the study, a total of 31 1906 SNP sites with mutated codon in 22 873 unigenes were discovered in both jute species (12 plants). Of which, 18 964 unigenes had SNP sites with mutated amino acid and the number of those for each unigene varied from 1 to 92 (Fig. 5A). Unigenes with such a SNP were the most common (3756) followed by those with two (2659), while there were only 7.5% unigenes with more than 15 SNP sites with mutated amino acid. In total, 546 DEGs with SNP sites resulted in amino acid change were discovered. The the most common DEGs (107) were those with a SNP with mutated amino acid (Fig. 5B).

### Validation of the differential gene expression

**Figure 6:**
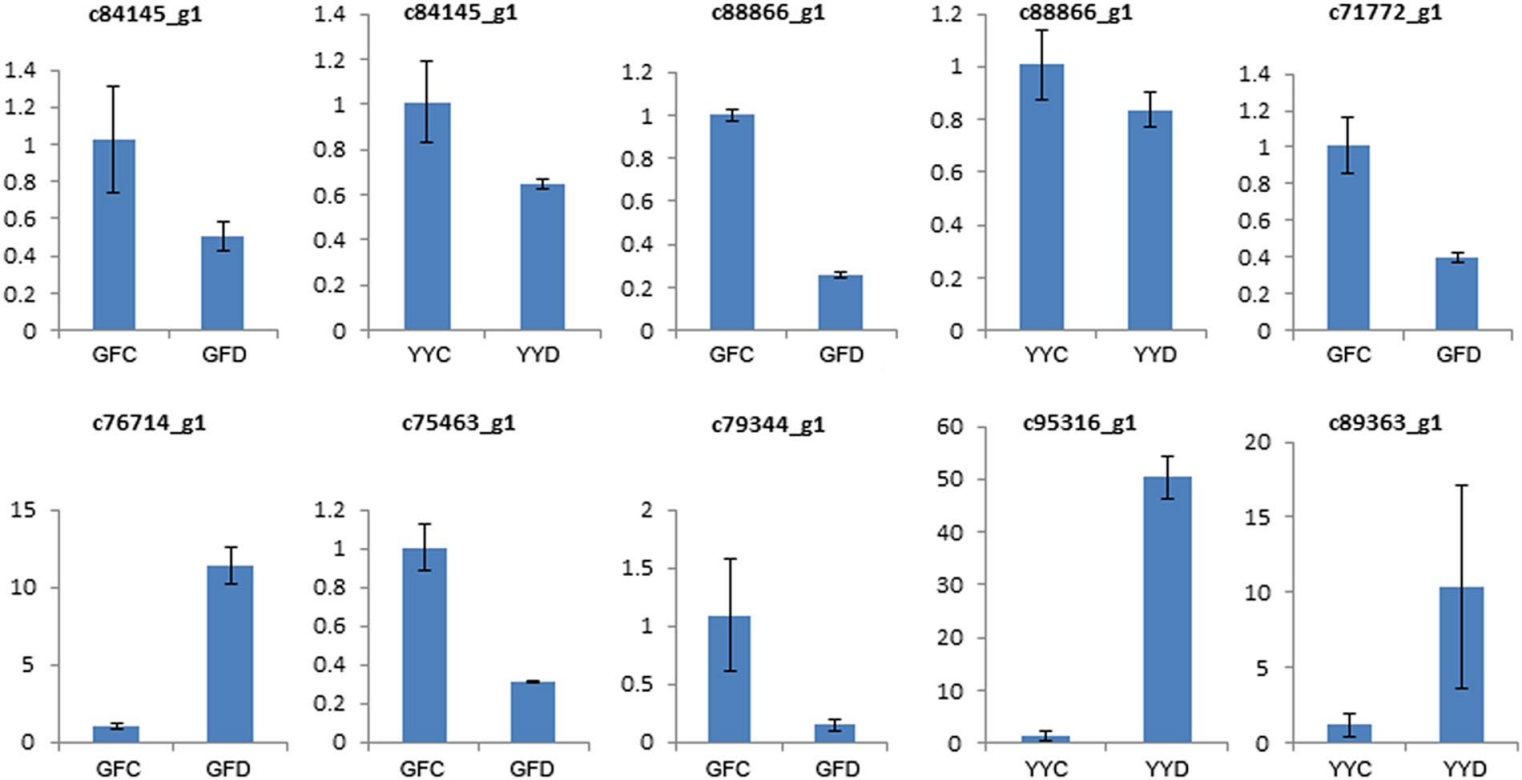
qRT-PCR analysis of eight differentially expressed unigenes. Data represent the fold change of relative quantification of each DEG in GFD (GF drought-stressed) vs. GFC (GF control) or YYD (YY drought-stressed) vs. YYC (YY control); the error bar represents the standard deviation.

To validate the differential expression results of the transcriptome sequencing data analysis, real-time quantitative PCR (qRT-PCR) was carried out in YY and GF under drought stress and control conditions. A total of eight DEGs were selected randomly for the qRT-PCR analysis. All the selected DEGs were significantly differentially expressed, and the expression profiles were consistent with the results of transcriptome sequencing. Fold change values of differential expression for each DEG obtained by qRT-PCR analysis in YY or GF are displayed in Fig. 6.

## Discussion

Drought is one of the major environmental factors affecting the geographical distribution of plants in nature. It causes significant yield loss in crop plants and threatens food security in many tropical and subtropical countries^24^. Therefore, it is essential to understand the molecular mechanisms of plant tolerance to drought to lay the foundation for crop molecular breeding. Jute (*Corchorus* spp.) is one of the most important bast fibre crops in the world. Although the jute genome^25^ have been published in this year, Yet, to our knowledge, to date, only two studies implementing transcriptome sequencing were conducted on jute (*C. capsularis*)^26,27^. The lack of gene sequences and functional annotation hindered the analysis of the genetic basis of molecular mechanisms triggered by abiotic stress in jute. In recent years, the accelerated development of sequencing platforms has made it possible to narrow the gap in our knowledge of the molecular mechanisms involved in abiotic stress in jute compared to those in other plants.

Overall, the number of detected DEGs was greater in YY than in GF, suggesting that the drought-sensitive jute species was relatively more vulnerable or hyper-responsive to drought stress at the molecular level. Similar results have been reported in studies on drought stress response in other plants^12,28,29^ and may be explained by the fact that compared to tolerant species, sensitive species undergo greater changes in phenotype, physiological and biochemical properties when mitigating the effects of stress conditions.

The GO annotation in GF showed DEG enrichment only in catalytic activity, which may contribute to the plant’s adaptation to drought stress through modifying the physiological and biochemical processes. Various environmental stresses such as drought and salt stresses can cause accumulation of reactive oxygen species (ROS), causing damages to cell wall and membrane tissues^24^. For example, ROS accumulation can cause crosslinking of phenolics and cell-wall glycoproteins, leading to cell-wall stiffening^30^. Antioxidant defence system plays a crucial role in scavenging ROS and protecting plants from oxidative damage. In YY, numerous DEGs were enriched in the antioxidant defence system terms: oxidation reduction process, oxidoreductase activity, peroxidase activity etc.; in addition, many pathways have participated in the complex regulatory and interaction network of ROS. The two pathways that are worth mentioning are phenylalanine metabolism and peroxidase pathways. Under drought stress, the leakage of photosynthetic electrons to the O2 is increased^31^; it is reported that the rate can reach to 50% as compared to unstressed in wheat^32^; which results in a mass of superoxide (O_2_^−^). And then ascorbate can function as an antioxidant directly to scavenge singlet oxygen and superoxide etc.^7,31^; while SOD (upregulated 104.42 fold change) can firstly dismutate superoxide to H2O2 which can promptly attacks thiol proteins^33^. Furtherly. CAT can scavenge H2O2 without a reductant^7^. And an increase of the activity of the main antioxidative systems (such as enzymes of the ascorbate-glutathione cycle) is generally accompanied by significant rise of activity and protein expression of NADP-ICDH^34^. The NADP-ICDH upregulated in YY can involve in thioredoxin reductase system and glutathione peroxidase system etc. to scavenge H_2_O_2_^10^. Phenolic acids are powerful antioxidants presented widely in plants. Of which, Ferulic acid (FA) and sinapic acid arising from the metabolism of phenylalanine have ability to quench ROS^11^. In particular, FA can enhance the activities of SOD and CAT to decrease content of ROS and involved in proline biosynthesis to regulate osmosis pressure under environment stress. And aldehyde dehydrogenase can synthesize FA and sinapic acid with coniferylaldehycle and sinapapaldehyde, respectively; while alcohol dehydrogenase can convert coniferylaldehycle and sinapapaldehyde into coniferylalcohol and sinapyl alcohol. However, in YY, c85671_g2 encoding aldehyde dehydrogenase was upregulated and c84589_g3 encoding alcohol dehydrogenase was downregulated, which might be conducive to deal with drought stress.

The primary plant cell wall consists of cellulose fibrils interconnected by hemicellulose tethers, such as arabinoxylan and xyloglucan, and embedded in pectin^24,30^. The plant cell wall undergoes extensive remodelling for survival under stress conditions^35^ to repair cell wall damage caused by ROS and maintain the osmotic pressure within the cell. The present study revealed the enrichment of 20 genes in the cell wall term: 17 were upregulated and 3 downregulated. Of these unigenes, 5 encoding for pectinesterase, which can remodel the cell wall, were significantly altered by drought stress in other plants^36,37^. In addition, the KEGG analysis revealed that many unigenes were also located in the biosynthesis pathways of various osmoprotectants, such as biosynthesis of amino acids, arginine and proline metabolism, and pentose and glucuronate interconversions, which support plant survival under different osmotic conditions, stabilise the membranes and proteins, and reduce the osmotic potential of membranes to prevent dehydration inside the cell^38,39^. Further, a large number of DEGs were located in the phenylpropanoid biosynthesis, citrate cycle, carbon fixation in photosynthetic organisms, and MAPK signalling pathways, which were enriched under various stresses and play crucial roles in plant survival under stress conditions^40–42^.

In the present study, 1769 unigenes were classified into 81 TF families. Of those, 34 DEGs from 20 TF families were found only in YY exposed to drought stress. A previous study^12^ on banana plants showed that the number of DEGs encoding TFs was higher in the sensitive genotype than in the tolerant genotype. The TF families, including MYB, AP2, bHLH, NAC, bZIP, C2C2-Dof, and HSFs, were differentially expressed under drought stress in the present study; these results were consistent with the results reported for other plants^3^. In addition, we found the largest number of DEGs (5) in the AP2-EREBP family, most of which were induced by drought stress; this family plays an important role in the regulation of transcription and signal transduction, such as phenylalanine metabolism and peroxidase pathways.

A large number of PK genes play an important role in phosphorylation events, which activate and deactivate the downstream signalling cascades under stresses^43^. For example, a study on *Arabidopsis* showed that the gene expression of some of the RLK members changes under water stress^44^. In the present study, 23 PK DEGs, including 19 RLK members, were identified in YY. Most of these DEGs were downregulated by drought stress, suggesting that the RLK members function as a negative regulator of drought tolerance in jute; our results were consistent with those reported by^45^ for rice.

With development of high-throughput sequencing, the molecular research of jute obtains a golden opportunity to grow up. However, compared with other crops, development of molecular markers and genes or QTLs mapping are still lacking because of a lag in molecular research on jute. To date, the densest genetic map includes only 913 polymorphic markers^46^, which greatly limits molecular marker-assisted selection and gene clones. Gene and QTL mapping involved in jute drought stress is not reported. In the study, The markers of SNP will be applicable in QTL and gene mapping in jute, particularly, the SNP sites located in DEGs can be used as developing markers which can be used directly as candidate drought-tolerance genes mapping or association analysis to improve efficiency of gene mapping.

In summary, we present the first comprehensive research on drought tolerance in two jute species, a drought-tolerant and a drought-sensitive species, exposed to drought stress conditions and elucidate the molecular basis of the drought tolerance mechanism. We identified 45,831 non-redundant unigenes and inferred the relationship between jute plants and other plant species based on unigene annotation by using molecular databases. The study analysed the DEGs in both jute species under drought stress condition and explored the GO terms and KEGG pathways related to drought tolerance. The results of the DEGs analysis showed that compared to the drought sensitive species, the drought tolerant species is less affected by drought stress. Further, 1769 TFs and 948 PKs were identified in the present study. Of these, 23 PK DEGs, including 19 RLK members, and 34 DEGs, encoding TFs from 20 TF families were detected in YY. Overall, we believe that the data presented herein will be useful to study the drought tolerance mechanism in plants, clone drought tolerance genes, and breed drought-tolerant jute cultivars.

## Materials and Methods

### Plant materials, drought stress treatment, and RNA isolation

Two jute species, drought-tolerant *C. olitorius* (Gangfengchangguo, GF) and drought-sensitive *C. capsularis* (Yueyuan5hao, YY), were used in this study. Sixty plants of each species were cultivated in a greenhouse at 25–28 °C in a hydroponic culture with Yoshida nutrient solution. Six disease-free seedlings at the 9-leaf stage, with strong and uniform growth, were selected from each species and equally divided into two groups: one was transferred to a control pot containing only Yoshida nutrient solution and the other was transferred to a pot containing a solution of equal parts of 10% polyethylene glycol and Yoshida nutrient solution (for drought treatment). After 24 h, the leaves and roots of every plant in the control and treatment pots were collected and used for RNA extraction.

Total RNA was isolated from the control and drought-stressed leaf and root samples using a Trizol reagent (Invitrogen, Carlsbad, CA, USA) according to the manufacturer’s instructions. The total RNA isolated from the leaves and from the root of a single plant was pooled in equal volume and concentration to prepare sequencing libraries. In total, twelve RNA sequencing libraries were prepared (three each GFD, GFC, YYD, and YYC; three independent biological replicates were prepared for each treatment of each species in this study). RNA degradation and contamination was monitored on 1% agarose gels, and RNA purity was checked using a NanoPhotometer^®^ spectrophotometer (Implen, Inc., Westlake Village, CA, USA). RNA concentration was measured using a Qubit^®^ RNA Assay Kit in a Qubit^®^ 2.0 Flurometer (Life Technologies, Carlsbad, CA, USA). RNA integrity was assessed using an RNA Nano 6000 Assay Kit and the Agilent Bioanalyzer 2100 system (Agilent Technologies, Santa Clara, CA, USA).

### Transcriptome sequencing

Sequencing libraries were generated using a NEBNext® Ultra™ RNA Library Prep Kit for Illumina® (New England Biolabs Inc., Ipswich, MA, USA) following the manufacturer’s recommendations, and index codes were incorporated to assign sequences to each sample. The quality of all libraries was assessed in the Agilent Bioanalyzer 2100 system. The clustering of the index-coded samples was performed on a cBot Cluster Generation System using a TruSeq PE Cluster Kit v3-cBot-HS (Illumina, USA) for RNA libraries according to the manufacturer’s instructions. After cluster generation, the library preparations were sequenced on an Illumina HiSeq X Ten platform and paired-end reads were generated for transcriptome sequencing.

### Transcriptome data analysis and annotation

Quality control of raw data was carried out using in-house perl scripts to remove reads containing adapter and poly-N sequences and low quality reads. All downstream analyses were based on clean, high-quality data. Transcriptome assembly for all clean data was accomplished using Trinity^47^ with min_kmer_cov set to 2 and all other parameters set to default. In addition, after obtaining the transcripts, all clean reads were mapped to the transcripts and the transcripts with less than 5X coverage were removed. Gene function was annotated based on the following public databases using the e-value cut-off listed parenthetically: Nr (e-value = 1e-5), Nt (e-value = 1e-5), Pfam (e-value = 0.01), KOG (e-value = 1e-5), Swiss-Prot (e-value = 1e-5), KO (e-value = 1e-10), and GO (e-value = 1e-6).

### Differential gene expression analysis and biological analysis of DEGs

Gene expression levels were estimated by RSEM^48^ for each sample. Clean data were mapped back onto the assembled transcriptome, and the read-count for each gene was obtained from the mapping results. Differential expression analysis of the two treatments groups of each species was performed using the DESeq R package (1.10.1)^49^. Genes with an adjusted P-value < 0.05 found by DESeq were assigned as differentially expressed. GO enrichment analysis of the DEGs was implemented by the GOseq R packages based on Wallenius non-central hyper-geometric distribution^50^. KOBAS^51^ software was used to test the statistical enrichment of DEGs in KEGG pathways.

### SNP calling

The assembled unigenes were used as reference transcriptome, and all of clean reads were mapped to the reference transcriptome. Identification of SNP refered to the method reported by Zhang *et al*.^27^. Raw vcf files were filtered with GATK^52^ standard filter method and parameters, and only SNPs with distance >5 were retained. The unigenes with SNP and differential expression were found out using in-house perl scripts.

### qRT-PCR analysis

To validate the results of the high-throughput sequencing, qRT-PCR of the same samples used for transcriptome sequencing was performed in an AB GeneAmp PCR System 9700 (Applied Biosystems, Foster City, CA, USA). The qRT-PCR was carried out in a two-step procedure according to the method by reported by Yangs^53^. The thermal cycle consisted of an initial denaturation at 95 °C for 10 min followed by 40 cycles at 95 °C for 10 s and 58 °C for 30 s. The relative expression levels were analysed according to a protocol described by Livak and Schmittgen^54^. Eight DEGs randomly selected from the RNA-seq results were used for validation; the jute *ELF* gene was selected as the endogenous control^55^. Each PCR reaction was conducted in triplicates. The primer sequence of DEGs and *ELF* gene are listed in Supplementary File 4.

## Electronic supplementary material

Supplementary Information

Supplementary file S1

Supplementary file S2

Supplementary file S3

Supplementary file S4
