# Supplementary material for: Transcriptome Analysis of Two Species of Jute in Response to Polyethylene Glycol (PEG)- induced Drought Stress
Source: Sci Rep. 2017 Nov 29;7:16565. doi: 10.1038/s41598-017-16812-5 (PMC5707433; doi:10.1038/s41598-017-16812-5)
Supplement: Supplementary file 1 — Supplementary Information [file 41598_2017_16812_MOESM1_ESM.docx]

Supplementary Infomation

**Transcriptome Analysis of Two Species of Jute in Response to Polyethylene Glycol (PEG)- induced Drought Stress**

Zemao Yang^1^, Zhigang Dai^1^, Ruike Lu^1^, Bibo Wu^2^, Qing Tang^1^, Ying Xu^1^, Chaohua Cheng^1^, Jianguang Su^1^*

**Supplementary file**

**Supplemental Figure 1.** Unigene annotation success rates across multiple databases.

**Supplemental Figure 2.** Gene ontology (GO) classification annotation of all unigenes.

**Supplemental Figure 3.** Kyoto Encyclopedia of Genes and Genomes (KEGG) Orthology (KO) annotation analysis of all unigenes.

**Supplemental Figure 4.** Eukaryotic ortholog group (KOG) annotation analysis of all unigenes.

**Supplementary file S1.** All differentially expressed unigenes (DEGs) in GFD (GF drought-stressed) vs. GFC (GF control) and YYD (YY drought-stressed) vs. YYC (YY control).

**Supplementary file S2.** Transcription factors (TFs) identified in this study.

**Supplementary file S3.** Protein kinases (PKs) identified in this study.

**Supplementary file S****4**. Primers for the DEGs and ELF used for qRT-PCR.


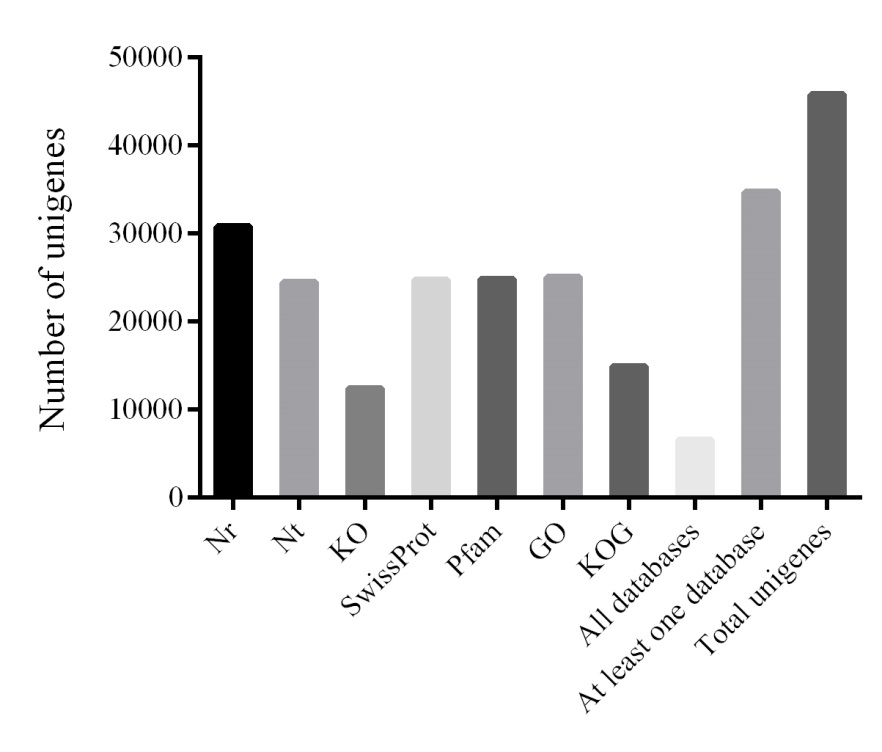


Supplemental Figure 1. Unigene annotation success rates across multiple databases.


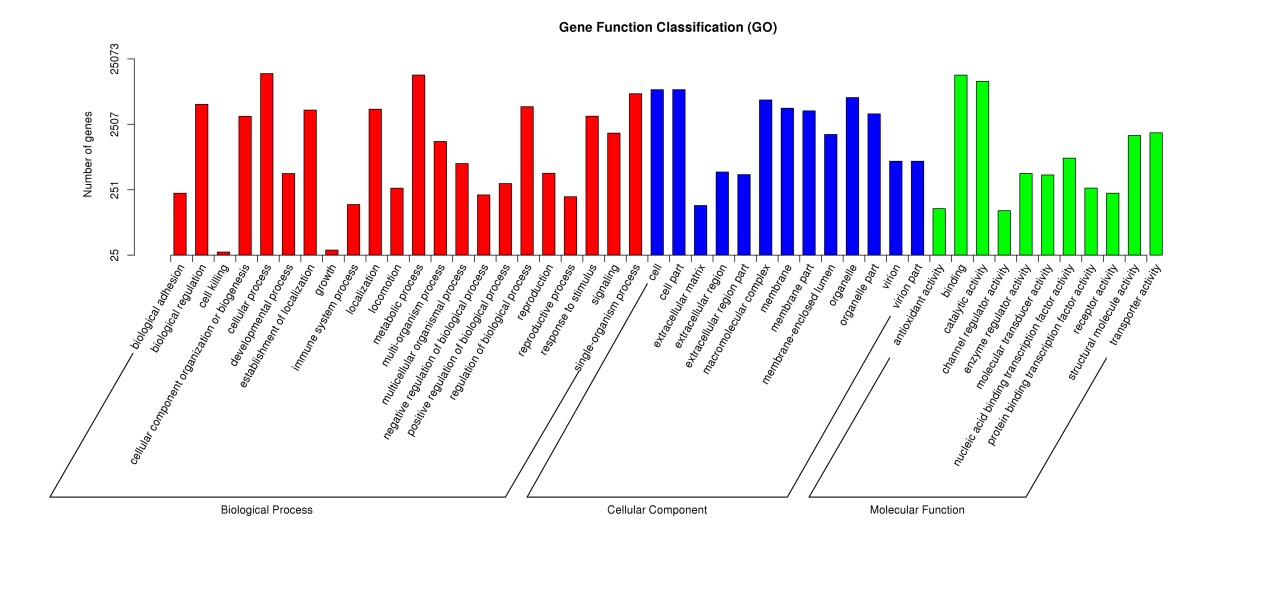


、**Supplemental Figure 2.** Gene ontology (GO) classification annotation of all unigenes.


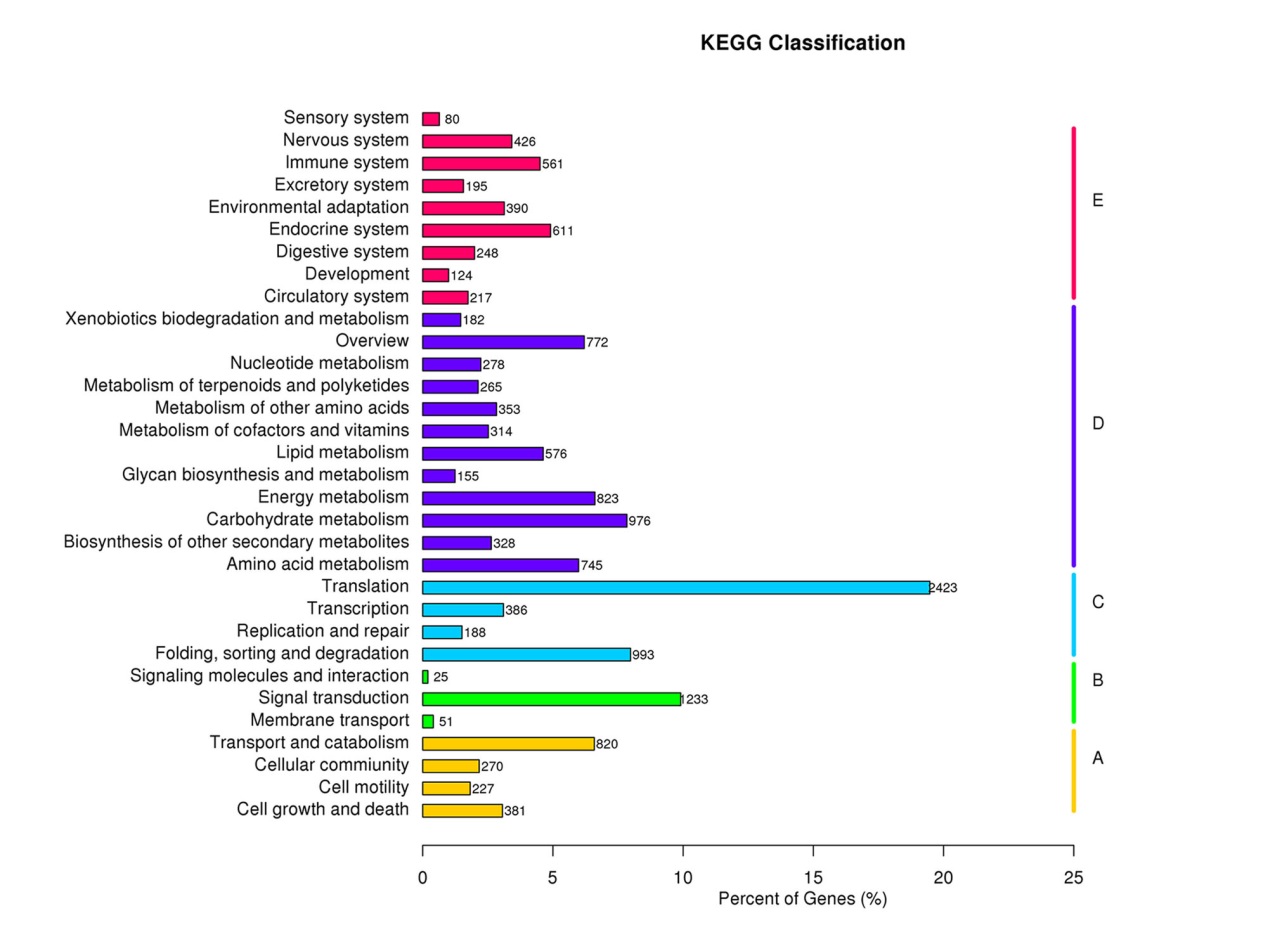


**Supplemental Figure 3.** Kyoto Encyclopedia of Genes and Genomes (KEGG) Orthology (KO) annotation analysis of all unigenes.


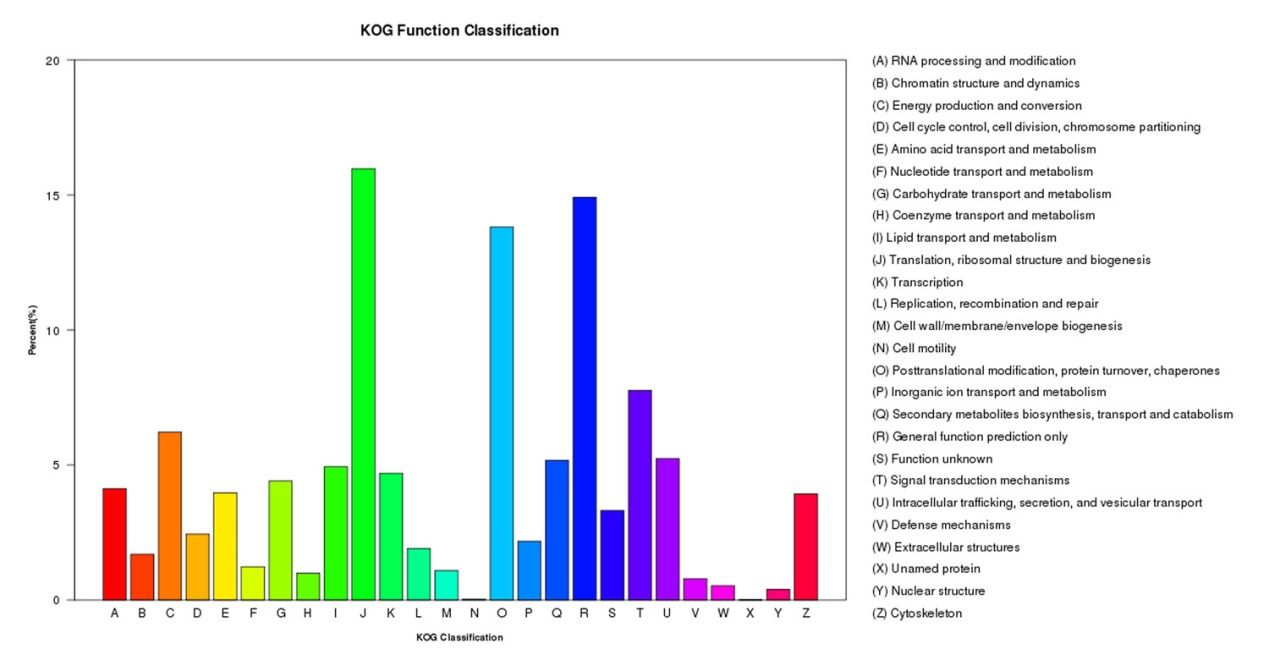


**Supplemental Figure 4.** Eukaryotic ortholog group (KOG) annotation analysis of all unigenes.
